# B cell activating factor (BAFF) levels are associated with failed remission after 12 months of treatment with benralizumab and mepolizumab in severe asthma

**DOI:** 10.1186/s12931-026-03500-0

**Published:** 2026-04-02

**Authors:** Bettina Corinna Fischer, Nora Drick, Bin Liu, Lennart Riemann, Anika Habener, David DeLuca, Anna-Maria Dittrich, Marius M. Hoeper, Hendrik Suhling, Gesine Hansen, Ruth Grychtol

**Affiliations:** 1 Department of Respiratory Medicine and Infectious Diseases, Hannover Medical School, Hannover, Germany; 2 Biomedical Research in Endstage and Obstructive Lung Disease Hannover (BREATH), Member of the German Center for Lung Research (DZL), Hannover, Germany; 3 Department of Pediatric Pneumology, Allergology and Neonatology, Hannover Medical School, Hannover, Germany; 4 Institute of Immunology, Hannover Medical School, Hannover, Germany; 5 Else-Kröner Fresenius Stiftung, Bad Homburg, Germany; 6 Excellence Cluster Resolving Infection Susceptibility RESIST (EXC 2155), German Research Foundation (DFG), Hannover Medical School, Hannover, Germany

**Keywords:** Severe eosinophilic asthma, Asthma remission, anti-IL-5 antibody, anti-IL-5 receptor antibody, Biomarkers, Benralizumab, Mepolizumab, Cytokines, Chemokines, Type 2 inflammation

## Abstract

The introduction of biologicals interfering with the interleukin-5 (IL-5) pathway has revolutionized the treatment of severe eosinophilic asthma (SEA), rendering remission an achievable goal even in patients with previously uncontrolled disease. This study investigated whether specific plasma biomarkers or clinical features could predict clinical remission in patients treated with an IL-5- (mepolizumab) or IL-5 receptor alpha-antibody (benralizumab) for severe eosinophilic asthma. Clinical remission, defined as sustained symptom control, absence of exacerbations, stable lung function, and no need for systemic corticosteroids, was assessed after 12 months. Plasma levels of 30 cytokines and chemokines were measured at baseline and after three months in 41 patients receiving either mepolizumab or benralizumab. Twelve patients (29%) achieved remission. Clinical characteristics and commonly used biomarkers, including blood eosinophil counts and fractional exhaled nitric oxide, showed no significant association with remission. However, elevated levels of B cell–activating factor (BAFF) at baseline and 3 months follow-up were significantly associated with failure to achieve remission. Logistic regression analyses revealed that plasma BAFF levels were predictive for remission status outperforming conventional eosinophilic inflammation markers. These findings suggest that elevated plasma BAFF may indicate B cell–driven inflammatory in severe eosinophilic asthma, which is not adequately addressed by therapies targeting interleukin-5. BAFF may serve as a valuable biomarker to identify patients less likely to respond to interleukin-5–directed treatments and who may benefit from alternative therapies. Further research is needed to validate these findings and explore the role of BAFF in personalized treatment strategies for severe asthma.

## Main text

Biologicals targeting Interleukin (IL) 5 (mepolizumab) or IL-5 receptor (benralizumab) are effective treatments for severe eosinophilic asthma, reducing exacerbation frequency and improving asthma control [1, 2]. These monoclonal antibodies have advanced asthma management significantly, rendering clinical remission an attainable treatment goal in patients with severe asthma [3]. Clinical characteristics associated with asthma remission have been studied, but biomarkers predicting outcome are still scarce [4].

In this real-life study, plasma chemokines and cytokines in patients with severe eosinophilic asthma were measured at baseline and three months after initiation of treatment with mepolizumab or benralizumab, aiming to identify biomarkers predictive of clinical outcome after one year. Patients with severe asthma were recruited at the respiratory clinic of Hannover Medical School. The study was conducted in accordance with the principles of the Declaration of Helsinki and approved by the local ethics committee (9170_BO_K_2020). All patients gave written informed consent.

Thirty plasma analytes were included in the analysis, based on at least 80% measurements within detection range: PAI-1, PTX3, sCD40L, sCD25, CXCL12, sST2, sTNF-R1, sTNF-RII, sRAGE, CX3CL1, gp130 (Biolegend #40775), APRIL, BAFF (Biolegend #740535), IL-12p40, IL-18 (Biolegend #740102), IL-5 (Biolegend #741027), RANTES, IL-8, CXCL10 (IP-10), Eotaxin-1, CCL17 (TARC), CCL2 (MCP-1), CCL3 (MIP-1a), CXCL9 (MIG), CXCL5 (ENA-78), CXCL1 (GROα), CXCL11 (I-TAC), CCL4 (MIP-1b) (Biolegend #740003), SLPI (R&D Quantikine ELISA DPI00), EDN (Nordic BioSite EKX-M44JTD-96). ELISAs and bead-based immunoassays were performed according to manufacturer’s instructions. Inferential statistics used ANOVA, Kruskal-Wallis, Mann-Whitney or t-Test as appropriate.

We used mixed effect models to interrogate whether cytokine levels at baseline and three months follow-up were associated with remission after one year. Each of the 30 cytokines was modelled individually as a log linear function of remission status and treatment and the additional covariates sex and oral steroid intake. The mixed effect model accounted for repeat measures by use of a patient-dependent random intercept. Multiple test correction across cytokines was done using the Benjamini-Hochberg method. Asthma remission was assessed after 12–16 months and defined as sustained absence of symptoms (Asthma Control Test (ACT) score ≥ 20 points), no exacerbations (asthma attacks requiring systemic glucocorticoid therapy for ≥ 3 consecutive days, or an increase in maintenance oral corticosteroid dose, or hospitalization/ emergency department visit), stable lung function (improved or unchanged FEV_1_), and no requirement for regular systemic glucocorticoidtreatment [3]. The study included 41 patients, with 66% (27/41) receiving benralizumab and 34% (14/41) mepolizumab. Overall, 12 patients (29%) achieved remission, eight in the benralizmab group (30%, 8/27) and four in the mepolizumab group (29%, 4/14). No significant differences (*p* ≥ 0.05) were observed between the remission and non-remission group regarding sex (male 42% vs. 58%), age (55.5 ± 8.5 vs. 53.4 ± 12.6 years), Body Mass Index (BMI) (27.9 ± 2.8 vs. 28.6 ± 6.5 kg/m²), number of exacerbations in the previous year (2.0 ± 1.6 vs. 2.7 ± 2.1), Asthma Control Test (13.4 ± 5.7 vs. 12.6 ± 4.5 points), mean blood eosinophils (603 ± 542/µl vs. 528 ± 541/µl), presence of allergy (50% vs. 57%), and forced expiratory volume in one second (FEV1) (z-score − 2.23 ± 1.4 vs. -2.20 ± 1.6).

**Fig. 1:**
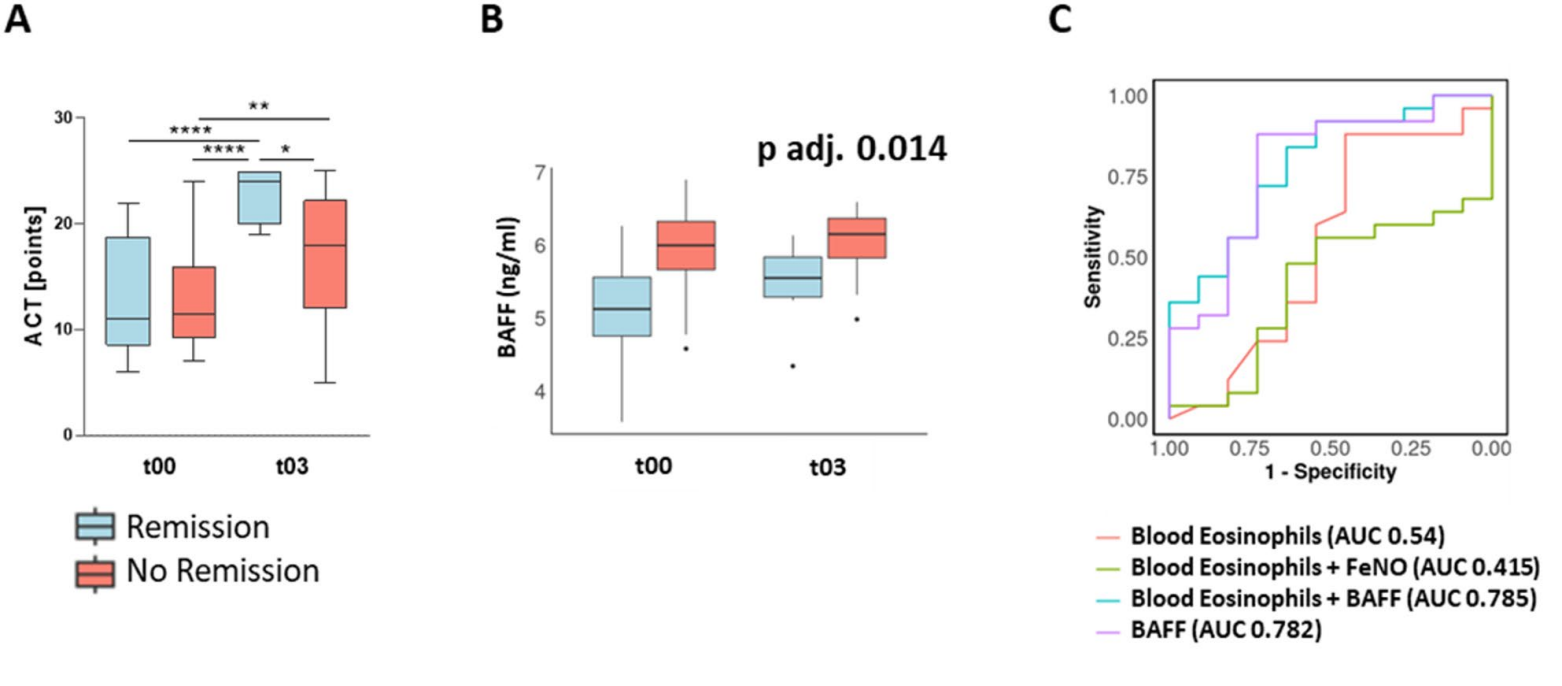
Change of ACT (**a**) was compared between baseline (t00) and three months follow-up (t03) using ANOVA. Increased BAFF levels were associated with failure to achieve remission after one year in mixed effect models interrogating 30 logarithmized plasma analytes measured at baseline and three months follow-up while correcting for oral steroid intake, sex and multiple testing. The adjusted *p*-value represents the overall result of the mixed effect models. Logarithmic BAFF levels are depicted (**b**). ROC-curves confirmed good predictive properties of BAFF for remission with no additive effect from blood eosinophils (**c**). ACT, Asthma control test; BAFF; B cell activating factor; adj, adjusted; ROC, Receiver operating characteristics. * *p* < 0.05, ** *p* < 0.01, **** *p* < 0.0001

Several readily available biomarkers and clinical characteristics were analyzed regarding their association with remission after one year. Baseline blood eosinophils, total immunoglobuline E (IgE), number of exacerbations, BMI, FEV1 z-score, and ACT were included in a mixed effect model, but no significant associations were found with remission after one year. Furthermore, changes in blood eosinophils, FEV1, ACT, or fractional exhaled nitric oxid (FeNO) from baseline and after three months showed no consistent differences between the remission and non-remission group with the exception of ACT scores, which improved significantly in both groups after three months but with greater increase in remission patients (Fig. 1a). Mixed-effect models were subsequently employed to assess changes in 30 plasma analytes between baseline and three months follow-up and their association with asthma remission after 12 months. In this model, remission after 12 months of treatment was associated with low BAFF levels at baseline and follow-up (p adj. = 0.014) (Fig. 1b). Additionally, CXCL10 and IL-18 were reduced in patients achieving remission, although this difference did not reach significance after multiple test correction (p unadj. = 0.034 and p unadj. = 0.045, respectively; data not shown). The only cytokines that showed significant changes from baseline to follow-up were IL-5 (p adj. = 1.48e-10) and eotaxin-1 (p adj. = 1.05e-5), however, these changes were not associated with remission.

To evaluate the predictive value of BAFF relative to eosinophilic inflammation markers for remission at one year, a series of logistic regression models were created and Receiver Operating Characteristic (ROC) curves were derived to assess predictive performance of response as a binary outcome. Blood eosinophils alone or in combination with FeNO were not predictive of remission (AUC = 0.54 and 0.415, respectively). In contrast, BAFF alone showed good predictive performance (AUC = 0.782), with marginal improvement when combined with blood eosinophils (AUC = 0.785) (Fig. 1c).

Our results identified BAFF as a potential biomarker for predicting failed asthma remission in patients treated with IL-5 targeting biologicals. Overall, biologicals targeting type 2 cytokines (IL-4, IL-5, IL-13) effectively treat severe type 2 asthma. IL-5 drives eosinophilic inflammation, IL-13 promotes airway hyperreactivity and mucus hypersecretion, and IL-4 triggers IgE class switch in allergic asthma. BAFF, part of the TNF superfamily, supports B cell maturation, survival and IL-4 induced IgE class switching [5].

Asthma patients have increased BAFF levels in plasma and sputum correlating with IgE and sputum B cells [6, 7]. BAFF is also upregulated in nasal polyps of chronic eosinophilic rhinosinusitis patients and correlates with IgE tissue levels and increased serum BAFF levels are predictive of recurrence of nasal polyps after surgical removal [8, 9]. Furthermore, sputum BAFF levels have not only been associated with IgE mediated airway inflammation but also with increased presence of autoantibodies in sputum of severe asthma patients with poor response to systemic corticosteroids potentially due to augmented eosinophil degranulation caused by autoantibodies [10].

Our results and published evidence suggest that elevated plasma BAFF reflects B cell activation and immunoglobulin-mediated inflammation, which anti-IL-5 biologics cannot address. In our cohort, BAFF levels were not linked to reported presence of allergies or total plasma IgE levels (data not shown), suggesting BAFF-associated inflammation is not solely tied to allergy. Furthermore, IgE is not only a marker for allergies, as studies have found increased tissue IgE in bronchial biopsies (asthma) and nasal polyps (CRS) regardless of atopy [11, 12]. Additionally, autoantibodies in the lungs of severe asthma patients suggest B cell-driven inflammation beyond allergies [10, 13]. Accordingly and supporting our findings, elevated BAFF levels have been linked to autoantibodies in sputum of severe eosinophilic asthma patients and failure of anti-Il5 directed therapies [14].

It remains unclear, whether patients who failed to achieve remission might respond more favourably to dupilumab, which targets the IL-4 receptor and dampens B cell class switch. Published data indeed indicate better response to dupilumab due to its multiple mechanisms addressing type 2 inflammation [15]. BAFF levels did not significantly change between baseline and follow-up in the remission group, although there was a trend for higher BAFF levels in the remission group at the three months follow-up. Further studies are needed to investigate the influence of IL-5 targeting drugs on BAFF levels and its utility for assessing treatment response over time. Notably, BAFF levels remained consistently increased in the non-remission group. Lastly, another limitation of our study is the lack of data about BAFF levels and immunoglobulin profiles in sputum to support our finding, exclusion of IL-4 and IL-13 and other analytes from the analysis due to poor assay performance and relatively small cohort size.

In summary, elevated plasma BAFF levels were associated with failure to achieve asthma remission after 12 months treatment with IL-5 targeting biologics. BAFF may represent a potential biomarker for the identification of patients with B cell–driven airway inflammation, potentially associated with allergic sensitisation or autoantibody-mediated immunopathology. Further studies need to confirm BAFF’s predictive value in personalized medicine and its potential to identify patients who may particularly benefit from IL-4 receptor blockers like dupilumab.

## Data Availability

The datasets generated and/or analysed during the current study are not publicly available due to privacy reasons but are available from the corresponding author on reasonable request.

## Abbreviations

IL-5: Interleukin-5
PAI-1: Plasminogen Activator Inhibitor-1
PTX3: Pentraxin-3
sCD40L: Soluble CD40 Ligand
sCD25: Soluble CD25
CXCL12: C-X-C Motif Chemokine Ligand 12
sST2: Soluble ST2
sTNF-R1: Soluble Tumor Necrosis Factor Receptor 1
sTNF-RII: Soluble Tumor Necrosis Factor Receptor 2
sRAGE: Soluble Receptor for Advanced Glycation Endproducts
CX3CL1: C-X3-C Motif Chemokine Ligand 1
gp130: Glycoprotein 130
APRIL: A Proliferation-Inducing Ligand
BAFF: B Cell-Activating Factor
IL-12p40: Interleukin-12p40
IL-18: Interleukin-18
IL-5: Interleukin-5
RANTES: Regulated upon Activation, Normal T Cell Expressed and Secreted
IL-8: Interleukin-8
CXCL10 (IP-10): C-X-C Motif Chemokine Ligand 10 (Interferon-Induced Protein 10)
Eotaxin-1: Eotaxin-1
CCL17 (TARC): C-C Motif Chemokine Ligand 17 (Thymus and Activation-Regulated Chemokine)
CCL2 (MCP-1): C-C Motif Chemokine Ligand 2 (Monocyte Chemoattractant Protein-1)
CCL3 (MIP-1a): C-C Motif Chemokine Ligand 3 (Macrophage Inflammatory Protein-1 alpha)
CXCL9 (MIG): C-X-C Motif Chemokine Ligand 9 (Monokine Induced by Gamma Interferon)
CXCL5 (ENA-78): C-X-C Motif Chemokine Ligand 5 (Epithelium Neutrophil-Activating Protein 78)
CXCL1 (GROα): C-X-C Motif Chemokine Ligand 1 (Growth-Regulated Oncogene alpha)
CXCL11 (I-TAC): C-X-C Motif Chemokine Ligand 11 (Interferon-Inducible T-cell Alpha Chemokine)
CCL4 (MIP-1b): C-C Motif Chemokine Ligand 4 (Macrophage Inflammatory Protein-1 beta)
SLPI: Secretory Leukocyte Protease Inhibitor
EDN: Eosinophil-Derived Neurotoxin
ANOVA: Analysis of Variance
Mann-Whitney: Mann-Whitney U Test
FeNO: Fractional Exhaled Nitric Oxide
AUC: Area Under the Curve
ROC: Receiver Operating Characteristic
ACT: Asthma Control Test
BMI: Body Mass Index
FEV1: Forced Expiratory Volume in 1 s
